# The correlation between triiodothyronine and the severity of liver fibrosis

**DOI:** 10.1186/s12902-022-01228-8

**Published:** 2022-12-12

**Authors:** Weiwei He, Caoxin Huang, Liying Wang, Weijuan Su, Shunhua Wang, Peiying Huang, Xiaofang Zhang, Yinxiang Huang, Yan Zhao, Mingzhu Lin, Xiulin Shi, Xuejun Li

**Affiliations:** 1 School of Medicine, Xiamen University, Xiamen, China; 2 Xiamen Diabetes Institute, The First Affiliated Hospital of Xiamen University, Xiamen, China; 3 Fujian Provincial Key Laboratory of Translational Medicine for Diabetes, Xiamen, Fujian China; 4 Department of Endocrinology and Diabetes, The First Affiliated Hospital, Xiamen University, No.55 Zhenhai Road, 361003 Xaimen, China

**Keywords:** Non-alcoholic fatty liver disease, Fibrosis, Type 2 diabetes mellitus, Free triiodothyronine

## Abstract

**Background:**

The severity of liver fibrosis is an important predictor of death in patients with non-alcoholic fatty liver disease (NAFLD) and type 2 diabetes mellitus (T2DM). However, there is still no definite conclusion on the relationship between triiodothyronine (T3) and the severity of liver fibrosis. Thus, the aim of this study was to analyze the correlation between T3 level and the severity of liver fibrosis.

**Methods:**

We performed a cross-sectional study of 2072 T2DM patients with normal thyroid function from January 2017 to January 2020. NAFLD fibrosis score (NFS), Fibrosis index based on the 4 factors (FIB-4) and BARD score (BARD) were used to assess the severity of fibrosis in T2DM patients, and linear regression analyses were used to determine the factors independently associated with liver fibrosis. Further experiments were performed to assess the impact of low T3 on fibrosis progression in mice model and explore possible mechanisms.

**Results:**

Free triiodothyronine (fT3) levels had significantly inverse correlations with NFS and FIB-4, and BARD in T2DM patients (*P* < 0.05). In multiple linear regression analyses, decreased fT3 level was an independent risk factor for the severity of liver fibrosis of T2DM patients (*P* < 0.01). Findings from in-vivo experiment using mice model proved that hypothyroidism mice had more severe of liver fibrosis than those mice with normal thyroid function. We also found that T3 could inhibit the profibrotic TREM2^+^CD9^+^ macrophage, which had been identified an important player in the progression of liver fibrosis.

**Conclusion:**

The findings from this study proved an inverse correlation between T3 level and the severity of liver fibrosis, and lower fT3 level within the normal range was an independent risk factor for severe liver fibrosis.

**Supplementary Information:**

The online version contains supplementary material available at 10.1186/s12902-022-01228-8.

## Introduction

Non-alcoholic fatty liver disease (NAFLD) is a clinicopathological syndrome characterized by hepatic steatosis [1]. It is a liver disease caused by other causes excluding alcohol, viral hepatitis, autoimmune liver disease and drugs, and has rapidly become a global public health problem [2]. NAFLD is a hepatic manifestation of the metabolic syndrome (MetS) and is also associated with many metabolic diseases including type 2 diabetes mellitus (T2DM), obesity, hyperlipidemia and arterial hypertension [3]. NAFLD is the most prevalent chronic liver disease worldwide until now, and its prevalence has been on the rise over the past decade, with a global prevalence of 25.24% and a prevalence of 27.37% in Asia [4]. NAFLD mainly includes non-alcoholic fatty liver (NAFL) and the more severe non-alcoholic steatohepatitis (NASH), which is characterized by hepatic steatosis and inflammation with or without fibrosis and can progress to cirrhosis or even hepatocellular carcinoma (HCC) [5].

NAFLD and T2DM are common diseases that often coexist and can act synergistically to cause adverse outcomes [6]. The coexistence of NAFLD and T2DM increases the likelihood of the onset of diabetic complications and increases the risk of progression of NAFLD to cirrhosis, HCC, and even death [7]. Studies have shown that almost 70% of diabetic patients may also have NAFLD, and the prevalence of biopsy-proven NASH is 20% in T2DM patients with normal liver function [8, 9]. The prevalence of prediabetes and T2DM is 85% in patients with NAFLD, and is more than two times higher than that in the general population [10]. Liver biopsy studies have clarified that the coexistence of NAFLD and T2DM has an absolute cumulative effect on cirrhosis, liver-related and all-cause mortality, and that the adverse consequences of the coexistence of NAFLD and T2DM are more severe than either condition alone [11, 12]. The American Diabetes Association (ADA) recommends that T2DM patients who have elevated plasma alanine aminotransferase (ALT) or steatosis of liver should be screened and evaluated for liver fibrosis and NASH, and that screening for NAFLD should focus on identifying patients with liver fibrosis, among whom early intervention may prevent progression to the decompensated cirrhotic stage [13]. The severity of liver fibrosis is an important predictor of cirrhosis, liver transplantation and liver-related death in patients with NAFLD, where the risk of liver-related death increases exponentially with increasing fibrosis stage [14]. Studies have shown that advanced liver fibrosis exists in 5-7% of T2DM patients and is even more common in obese T2DM patients, whose prognosis is usually poor and need early intervention to reduce fibrosis progression [15–17]. However, the pathogenesis of hepatic fibrosis progression is not fully understood and requires urgent in-depth study.

Thyroid hormones (THs) mainly include thyroxine (T4) and triiodothyronine (T3) [18]. T4 can become biologically active T3 by deiodination with type I and II deiodinases, and free triiodothyronine (fT3) is the most sensitive indicator of thyroid function [19]. Hypothyroidism patients have lower THs such as lower free thyroxine (fT4) and lower fT3 than euthyroid individuals. Hypothyroidism is now well established as an important risk factor for the development of NAFLD [20–22]. In addition, several studies have demonstrated that hypothyroidism and subclinical hypothyroidism are also important in promoting NASH and liver fibrosis progression [23–26]. A cross-sectional study that included 425 patients with biopsy-proven NAFLD showed that hypothyroid patients had 2-fold risk of advanced liver fibrosis compared to those with normal thyroid function, suggesting that hypothyroidism was an independent risk factor for advanced fibrosis [27]. Another study showed that the lower fT3 level, the greater likelihood of patients developing advanced NASH fibrosis, further demonstrating fT3 as a key factor of influencing the progression of fibrosis in patients with NAFLD [26]. However, there was still no definite conclusion on the relationship between T3 and the severity of liver fibrosis and the mechanism was unclear. Thus, we analyzed the correlation between fT3 levels and liver fibrosis severity in T2DM patients through a cross-sectional study. In addition, we assessed the impact of hypothyroidism on progression of liver fibrosis in mice model of NASH, and explored the possible mechanism via cell culture.

## Methods

### Study design

This cross-sectional study was conducted at the First Affiliated Hospital of Xiamen University from January 2017 to January 2020. The study was approved by the Ethics Committee of the First Affiliated Hospital of Xiamen University, and all patients gave informed consent. This study was conducted in accordance with the fundamental principles of the Declaration of Helsinki. All participants were inpatients with definite diagnosis of T2DM in the Department of Endocrinology and Diabetes. Inclusion criteria for this study were as following: (1) Patients with T2DM; 2) No less than 18 years; 3) With enough data to evaluate thyroid functions and liver fibrosis severity. Exclusion criteria for this study were as following: (1) age < 18 years; (2) non-T2DM patients; (3) heavy alcohol consumption (> 20 g/day for women and > 30 g/day for men); (4) liver disease due to virus, alcohol, drugs, autoimmunity and/or total parenteral nutrition; (5) without complete information such as thyroid function; (6) patients with overt thyroid diseases or abnormal thyroid function.

### Laboratory testing

Inpatients were fasted overnight for 8 h. Fasting plasma glucose (FPG), triglycerides (TG), total cholesterol (TC), triacylglyceride (TG), high density lipoprotein cholesterol (HDL-C), low density lipoproteincholesterol (LDL-C), and ALT, aspartate aminotransferase (AST), serum creatinine (sCr), blood urea nitrogen (BUN), serum uric acid (SUA) and total bilirubin (TBIL) were measured by fasting blood sampling the next morning. fT3, fT4 and thyroid-stimulating hormone (TSH) were measured by immunoradiometric. Normal values for fT3 range from 3.5 to 6.5 pmol/L, normal values for fT4 range from 11.5 to 22.7 pmol/L, and normal values for TSH range from 0.55 to 4.78 mIU/L. The diagnostic criteria for normal thyroid function were 3.5 < fT3 < 6.5 pmol/L, 11.5 < fT4 < 22.7 pmol/L, and 0.55 < TSH < 4.78 mIU/L, the diagnostic criteria for hyperthyroidism were increased fT4 and/or fT3 with TSH < 0.55 mIU/L, and the diagnostic criteria for hypothyroidism were decreased fT4 and/or fT3 with TSH > 4.78 mIU/L.

### Data collection

Data such as age, gender, body mass index (BMI), previous disease history and family history, as well as biochemical parameters including FPG, ALT, AST, TBIL, TG, TC, HDL-C, LDL-C, BUN, SUA and sCr were collected. Other key biochemical parameters such as thyroid hormone levels including TSH, fT4 and fT3 were collected.

### Liver fibrosis score

We assessed the severity of fibrosis in T2DM patients by three different fibrosis assessment methods: NAFLD fibrosis score (NFS), Fibrosis index based on the 4 factors (FIB-4) and the BMI, AST/ALT ratio and Diabetes (BARD) score (Supplementary Table 1) [28, 29].

### Experimental animals

Eight-week-old SPF C57BL/6J mice were housed at 21-23 °C, 55-60% humidity, and 12 h light/dark cycle with free access to water. Mice were purchased and acclimatized for 2 weeks, after which they were randomly divided into 3 groups: (1) High-fat with methionine and choline deficiency (HFMCD)-induced NASH group; (2) HFMCD-induced NASH + Propylthiouracil (PTU)-induced hypothyroidism group; (3) control group. The HFMCD-induced NASH model group was fed a high-fat and choline methionine-deficient diet (A06071301B16; Research Diets, New Brunswick NJ); the HFMCD-induced NASH + PTU-induced hypothyroidism group was fed with PTU (0.15%, P3755; Sigma) and the HFMCD diet; the control mice were fed with normal diet. The experiment lasted for 10 weeks, and the mice were sacrificed at 18 weeks of age.

### Histological staining

Freshly removed mouse liver tissues were immersed in fixative solution and fixed at room temperature for 12 h. After washing with 75% ethanol to remove the yellow color, the tissues were dehydrated with gradient ethanol, clear in xylene, waxed and embedded. The embedded tissue was sectioned using a microtome and then stained according to the Masson staining kit instructions. After sealing with neutral gum and observed under the microscope, the cytoplasm and muscle fibers were stained red, while the collagen fibers were blue. Paraffin-embedded sections were stained with Hematoxylin-Eosin (HE), and histopathological changes in the liver of each group of mice were observed with Motic VM1 microscope (McAudi, Hong Kong, China). Paraffin sections of mice liver were immunostained with anti-a-SMA (1:200, 72026T, CST) and Collagen 1 (1:200, ab5694, abcam) for immunohistochemistry. Adobe Photoshop (Adobe, San Jose, CA, USA) was used to edit digital photographs, and Image J (National Institutes of Health, Bethesda, MD, USA) was used for quantitative analysis.

### Peripheral blood mononuclear cells (PBMCs) isolation and culture

We collected 5mL of heparin sodium anticoagulated peripheral blood from a volunteer and used density gradient centrifugation to isolate PBMCs for subsequent cell culture. Isolated PBMCs were plated into fibronectin-coated cell culture dishes and subsequent experiments were conducted 24 h later. PBMCs were cultured in 1640 medium supplemented with 10% fetal bovine serum. The cells were cultured in culture plates at 37 °C and 5% CO_2_ in an incubator. To differentiate monocytes into macrophages, PBMCs were grown in 1640/FBS medium containing 100nM Phorbol-12-Myristate-13-Acetate (PMA) with a 2-day changing medium, during which PBMCs were treated with 100ng/mL T3 or Dimethyl sulfoxide (DMSO), and subsequent flow cytometry was performed after 6 days of culture.

### Flow cytometry

FITC anti-CD14^+^ cell antibody (Biolegend, 367,116), PerCP-Cy5.5-anti-CD9^+^ cell antibody (Biolegend, 312,110), PE/Cy7 anti-CSF1R antibody (Biolegend, 347,308) and APC-TREM2^+^ cell antibody (R&D Systems, FAB17291A) were used to analyze the ratio of CD9^+^TREM2^+^ cells of macrophages in PBMCs of T3-treated samples and DMSO-treated controls. The Quanteon flow cytometer (ACEA Biosciences) was used to perform flow cytometry.

### Statistical analysis

Continuous variables were expressed as mean with standard deviation (SD) and categorical variables were expressed as frequencies with percentages. Independent t-test or ANOVA test was used for continuous variables, and a chi-square test was used by us for categorical variables to assess the statistical differences between groups. To assess whether thyroid function was independently associated with liver fibrosis, we divided subjects into 3 groups according to TSH, fT3, and fT4 levels from low to high, and analyzed the correlation between the severity of liver fibrosis and the changes of THs levels. The independent impact of THs on liver fibrosis progression was investigated using multivariate logistic regression analysis. All statistical analyses were performed using STATA (Version 12.0). Statistical significance was defined as a *P* value < 0.05.

## Results

### Clinical cross-sectional study

#### Characteristics of included patients

A total of 2072 T2DM patients with normal thyroid function were included. The mean age of the included patients was 56.2 ± 13.8 years, and 1269 (61.24%) of them were male. Among the included individuals, 898 patients had hypertension and 343 patients were obese (BMI > 28). The mean levels of fT3, fT4 and TSH were 4.6 ± 0.5 pmol/L, 16.7 ± 2.2 pmol/L, and 1.7 ± 0.9 mIU/L, respectively. The mean NFS, FIB-4 score and BARD score was − 1.2 ± 1.2, 1.1 ± 0.8 and 2.3 ± 1.0, respectively.

#### Correlation between thyroid hormone levels and the severity of liver fibrosis in euthyroid T2DM patients

**Fig. 1:**
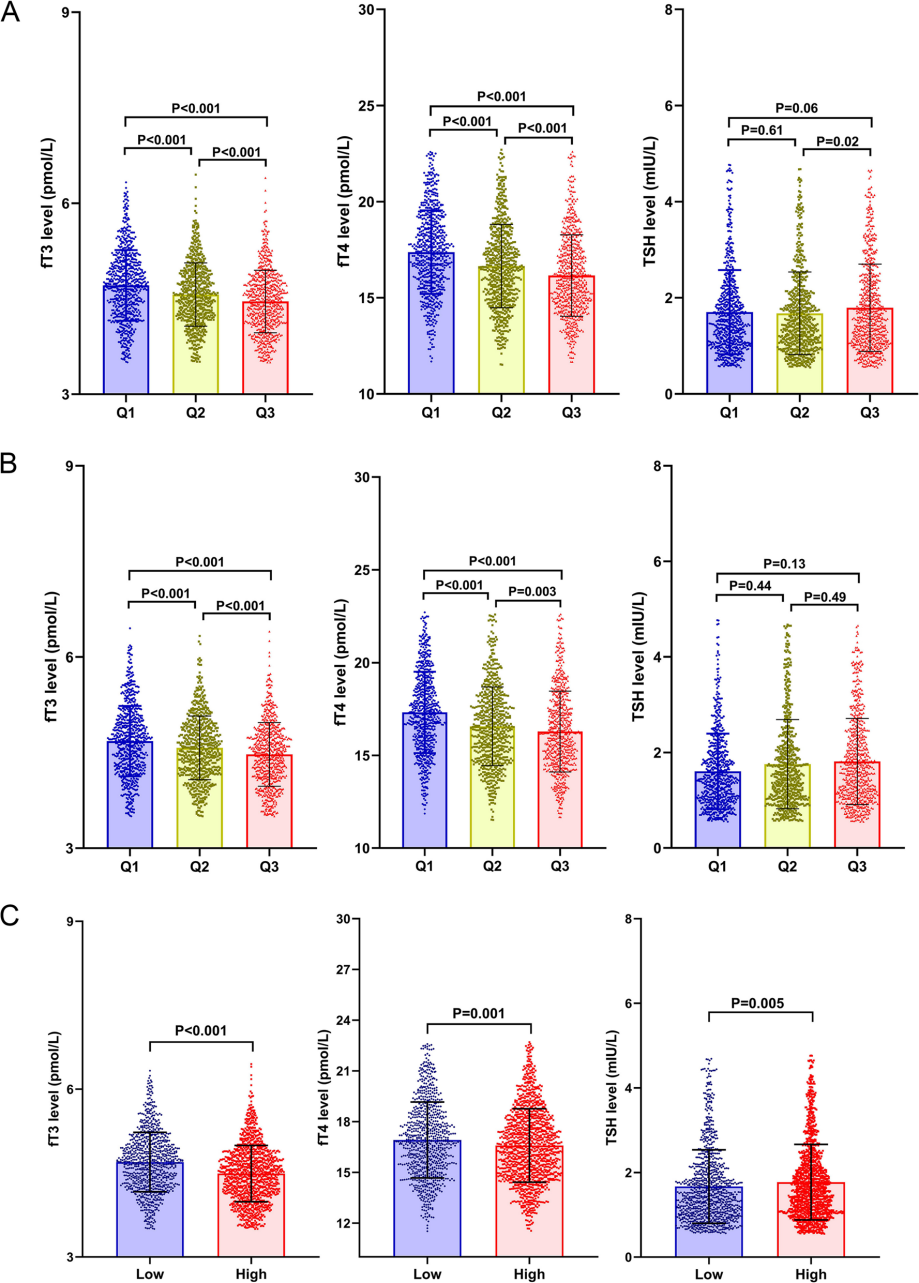
Changes in fT3, fT4, and TSH levels in the groups classified by the severity of liver fibrosis according to NFS, FIB-4 and BARD (A. Changes in fT3, fT4, and TSH levels among the groups classified by NFS; B. Changes in fT3, fT4, and TSH levels among the groups classified by FIB-4; C. Changes in fT3, fT4, and TSH levels among the groups classified by BARD. Patients were divided into 3 groups according to NFS, FIB-4 and BARD score from low to high, and differences between groups were analyzed.)

**Fig. 2:**
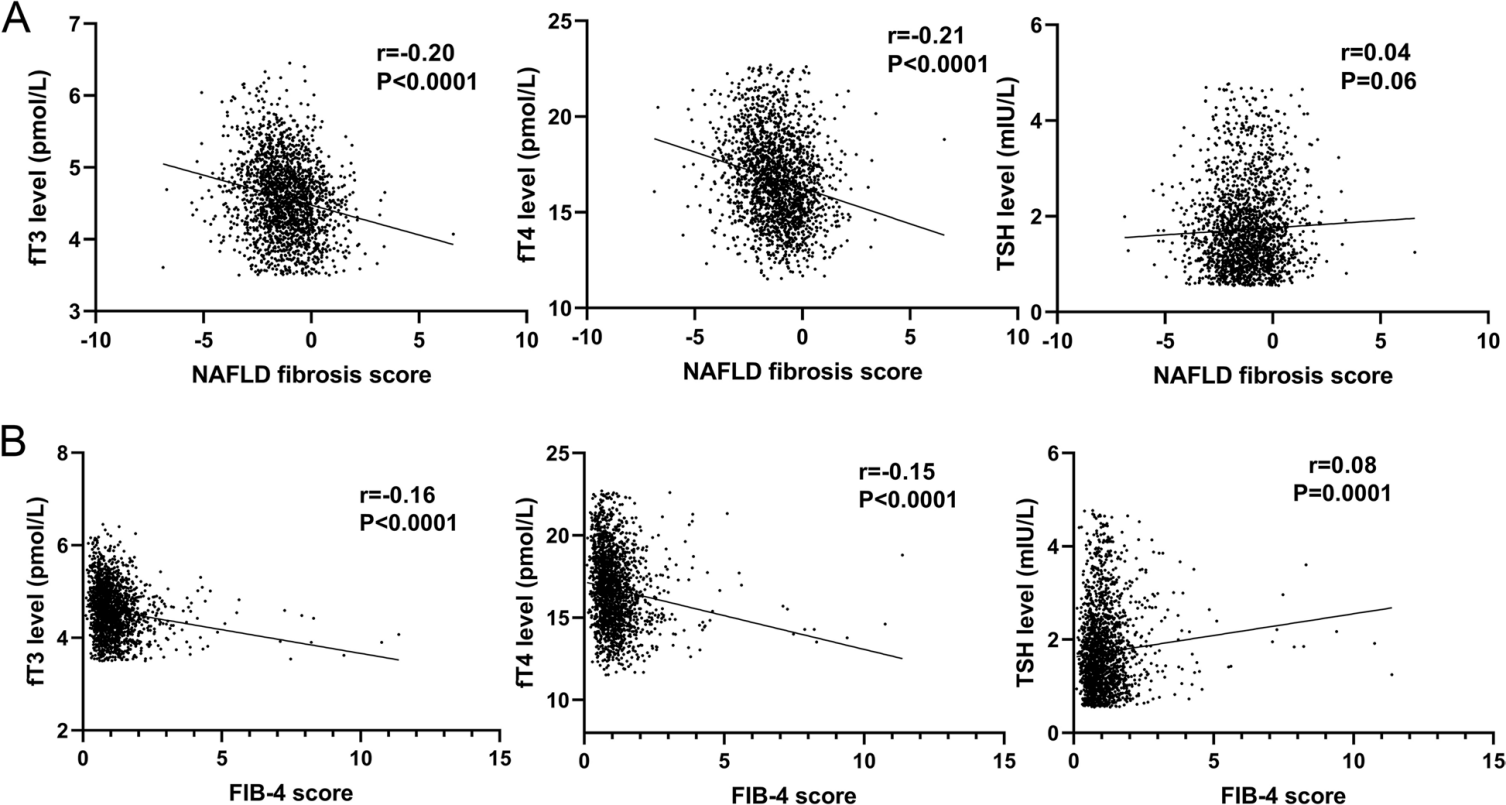
Linear correlation analyses of liver fibrosis scores with fT3, fT4 and TSH levels (A. Linear correlation analyses of NAFLD fibrosis score (NFS) with fT3, fT4 and TSH levels; B. Linear correlation analyses of FIB-4 score with fT3, fT4 and TSH levels. All T2DM patients were included, and the correlation coefficient r and corresponding P value were shown in the figure.)

Euthyroid T2DM patients were classified into 3 groups according to the risk of fibrosis by NFS, FIB-4 score or BARD score. The results showed that fT3 levels were significantly different among those groups classified by NFS, FIB-4 or BARD, and fT3 and fT4 levels were lower in the high-risk group than in the low-risk group (Fig. 1; Tables 1 and 2, Supplementary Table 2). We also found a significantly inverse correlation between fT3 levels and NFS and FIB-4 by linear correlation analyses (*r*=-0.20, *P* < 0.001; *r*=-0.16, *P* < 0.001); fT4 levels also had an inverse linear correlation with NFS and FIB-4 (*r*=-0.21, *P* < 0.001; *r*=-0.15, *P* < 0.001); TSH levels were only positively correlated with FIB-4 (*r* = 0.08, *P* = 0.001) (Fig. 2).

**Table 1:** Differences in the clinical features among those groups classified by NFS

Parameters	Q1	Q2	Q3	*P* values
Number	690	691	691	
Gender (Male, %)	418(60.6%)	424(61.4%)	427(61.8%)	0.896
Age (Year, Mean ± SD)	46.75(12.96)	55.67(11.45)	64.72(10.61)	< 0.001
Hypertension (%)	213(30.9%)	292(42.2%)	393(56.9%)	< 0.001
BMI (kg/M2, Mean ± SD)	24.00(3.76)	24.57(3.54)	25.52(4.55)	< 0.001
Obesity (%)	87(12.6%)	105(15.2%)	151(21.8%)	< 0.001
Duration (Year, Mean ± SD)	7.60(2.16)	7.95(3.91)	8.36(2.97)	< 0.001
TSH (mIU/L, Mean ± SD)	1.70(0.88)	1.68(0.86)	1.79(0.91)	0.066
fT4 (pmol/L, Mean ± SD)	17.37(2.17)	16.65(2.17)	16.16(2.12)	< 0.001
fT3 (pmol/L, Mean ± SD)	4.71(0.56)	4.57(0.50)	4.46(0.49)	< 0.001

*BMI B*ody mass index, *TSH* Thyroid stimulating hormone, *fT3* Free triiodothyronine, *fT4* Free thyroxine

**Table 2:** Differences in the clinical features among those groups classified by FIB-4 Score

Parameters	Q1	Q2	Q3	*P* values
Number	690	691	691	
Gender (Male, %)	404(58.6%)	415(65.3%)	404(58.5%)	0.795
Age (Year, Mean ± SD)	45.13(12.24)	57.44(10.49)	64.57(10.94)	< 0.001
Hypertension (%)	205(29.7%)	319(46.2%)	374(54.1%)	< 0.001
BMI (kg/M2, Mean ± SD)	24.84(3.90)	24.75(4.33)	24.50(3.81)	0.166
Obesity (%)	121(17.5%)	113(16.4%)	109(15.8%)	0.668
Duration (Year, Mean ± SD)	7.56(2.19)	8.14(3.96)	8.20(2.90)	< 0.001
TSH (mIU/L, Mean ± SD)	1.60(0.79)	1.76(0.93)	1.81(0.90)	< 0.001
fT4 (pmol/L, Mean ± SD)	17.32(2.19)	16.57(2.12)	16.29(2.18)	< 0.001
fT3 (pmol/L, Mean ± SD)	4.68(0.55)	4.58(0.50)	4.47(0.50)	< 0.001

*BMI* Body mass index, *TSH* Thyroid stimulating hormone, *fT3* Free triiodothyronine, *fT4* Free thyroxine

We also divided patients into three groups according to fT3 levels from low to high. The results showed that NFS, FIB-4 and BARD all decreased significantly with increasing fT3 levels (*P* < 0.001) (Supplementary Table 3).

#### **Reduced fT3 level was an independent risk factor for the severity of liver fibrosis in euthyroid T2DM patients**

Multiple linear regression analyses were used to further explore key risk factors of influencing the severity of liver fibrosis. We found that decreased fT3 level was an independent risk factor of liver fibrosis. After adjusting for confounders such as blood pressure, UA, and glucose, fT3 levels were significantly and negatively correlated with NFS, FIB-4, and BARD scores (*P* < 0.001); fT4 levels were negatively correlated with NFS and FIB-4 (*P* < 0.001), but not with BARD (*P* = 0.491) (Tables 3, 4 and 5). After dividing the patients into three groups (< 45 years, 45–65 years, > 65 years), linear regression analyses between fT3 and BARD scores were performed among patients in each age group. The results of univariate linear regression analysis showed a significant correlation between fT3 levels and BARD scores in both patients of < 45 years and 45–65 years (*P* = 0.002, *P* < 0.0001). A marginally significant correlation between fT3 levels and BARD scores in patients of > 65 years was observed (*P* = 0.068). The results of multiple linear regression analyses showed that fT3 levels were independently correlated with BARD scores in patients aged < 45 and 45–65 years after adjustment for SBP, DBP, duration, Cr, fT4, TSH and UA (*P* = 0.007, *P* = 0.005). The above results suggested reduced fT3 levels as an independent risk factor of liver fibrosis in T2DM patients with normal thyroid function, and that patients with higher fT3 levels within the normal range were at lower risk for liver fibrosis.

**Table 3:** Multiple linear regression analyses of factors associated with NFS

Parameters	NAFLD fibrisos score			
	Univariate linear regression		Multiple linear regression	
	Β (95% CI)	*P*	Β (95% CI)	*P*
Duration (Year)	0.033(0.016 ~ 0.050)	< 0.001	0.015(-0.001 ~ 0.032)	0.058
TSH (mIU/L)	0.059(-0.002 ~ 0.120)	0.056	-	-
fT4 (pmol/L)	-0.118(-0.142~ -0.095)	< 0.001	-0.088(-0.112~ -0.064)	< 0.001
fT3 (pmol/L)	-0.466(-0.566~ -0.366)	< 0.001	-0.199(-0.304~ -0.094)	< 0.001
SBP	0.005(0.002 ~ 0.008)	< 0.001	0.011(0.008 ~ 0.015)	< 0.001
DBP	-0.019(-0.024~ -0.014)	< 0.001	-0.026(-0.032~-0.020)	< 0.001
Cr	0.005(0.004 ~ 0.007)	< 0.001	0.004(0.002 ~ 0.006)	< 0.001
Mean glucose	0.106(0.077 ~ 0.136)	< 0.001	0.073(0.044 ~ 0.102)	< 0.001
UA	0.001(-0.001 ~ 0.001)	0.058	-	-

*TSH* Thyroid stimulating hormone, *fT3* Free triiodothyronine, *fT4* Free thyroxine, systolic blood pressure, *DBP D*iastolic blood pressure, *Cr C*reatinine, *UA* Uric acid

**Table 4:** Multiple linear regression analyses of factors associated with FIB-4 Score

Parameters	FIB-4 score			
	Univariate linear regression		Multiple linear regression	
	Β (95% CI)	*P*	Β (95% CI)	*P*
Duration (Year)	0.013(0.002 ~ 0.024)	0.024	0.005(-0.006 ~ 0.016)	0.377
TSH (mIU/L)	0.076(0.037 ~ 0.115)	< 0.001	0.058(0.018 ~ 0.099)	0.005
fT4 (pmol/L)	-0.053(-0.069~ -0.038)	< 0.001	-0.037(-0.054~ -0.021)	< 0.001
fT3 (pmol/L)	-0.236(-0.301~ -0.172)	< 0.001	-0.144(-0.216~ -0.073)	< 0.001
SBP	0.001(-0.001 ~ 0.003)	0.331	-	-
DBP	-0.009(-0.012~ -0.006)	< 0.001	-0.007(-0.010~ -0.004)	< 0.001
Cr	0.002(0.001 ~ 0.003)	< 0.001	0.001(0.0005 ~ 0.002)	0.003
Mean glucose	0.050(0.030 ~ 0.069)	< 0.001	0.037(0.017 ~ 0.057)	< 0.001
UA	0.0002(-0.0001 ~ 0.001)	0.148	-	-

*TSH T*hyroid stimulating hormone, *fT3* Free triiodothyronine, *fT4* Free thyroxine, Systolic blood pressure, *DBP* Diastolic blood pressure, *Cr* Creatinine, *UA* Uric acid

**Table 5:** Multiple linear regression analyses of factors associated with BARD Score

Parameters	BARD Score			
	Univariate linear regression		Multiple linear regression	
	Β (95% CI)	*P*	Β (95% CI)	*P*
Age (Year)	0.016(0.013 ~ 0.019)	< 0.001	0.013(0.009 ~ 0.017)	< 0.001
Duration (Year)	0.009(-0.005 ~ 0.023)	0.196	-	-
TSH (mIU/L)	0.083(0.033 ~ 0.132)	0.001	0.073(0.023 ~ 0.123)	0.004
fT4 (pmol/L)	-0.032(-0.052~ -0.013)	0.001	0.007(-0.014 ~ 0.028)	0.491
fT3 (pmol/L)	-0.306(-0.388~ -0.224)	< 0.001	-0.176(-0.265~ -0.086)	< 0.001
SBP	0.003(0.0003 ~ 0.005)	0.022	-0.0001(-0.003 ~ 0.003)	0.930
DBP	-0.006(-0.010~ -0.002)	0.004	-0.002(-0.007 ~ 0.004)	0.596
Cr	0.002(0.001 ~ 0.004)	< 0.001	0.001(0.0003 ~ 0.003)	0.011
UA	0.0003(-0.0002 ~ 0.0007)	0.216	-	-

*TSH* Thyroid stimulating hormone, *fT3* Free triiodothyronine, *fT4* Free thyroxine, Systolic blood pressure, *DBP* Diastolic blood pressure, *Cr* Creatinine, *UA* Uric acid

#### Low T3 levels exacerbated liver fibrosis in NASH mice and T3 inhibited the profibrotic macrophages

**Fig. 3:**
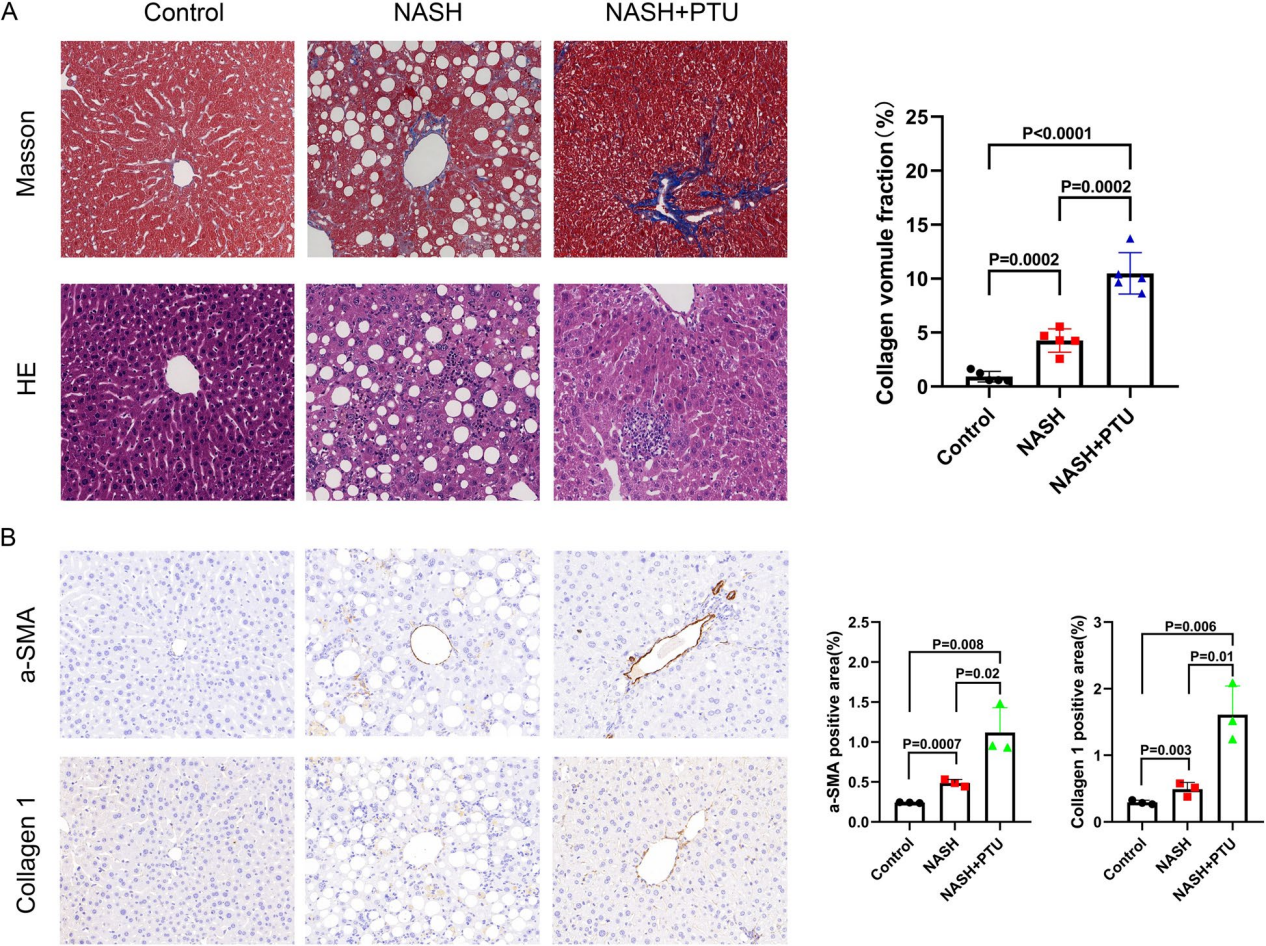
Experiment using mice model showed that T3 deficiency may promote liver fibrosis progression (**A**. Representative images of liver sections stained by HE and Masson staining from control, NASH and NASH + PTU mice (original magnification, ×200); **B**. Immunohistochemistry of a-SMA and Collagen 1 expression in each group.)

Through animal model studies, we found that hypothyroidism exacerbated the severity of liver fibrosis in NASH mice, and hypothyroidism mice had more severe of liver fibrosis than those mice with normal thyroid function (Fig. 3-A). The results of collagen volume fraction detected by Masson staining showed that the severity of liver fibrosis was highest in NASH mice with hypothyroidism compared to the control and NASH model mice (*P* < 0.0001, *P* = 0.0002) (Fig. 3-A). In addition, we found that liver immunostaining for a-SMA and collagen 1 was increased in NASH mice with hypothyroidism (Fig. 3-B). The above results suggested that reduced T3 may promote the progression of liver fibrosis in NASH mice.

**Fig. 4:**
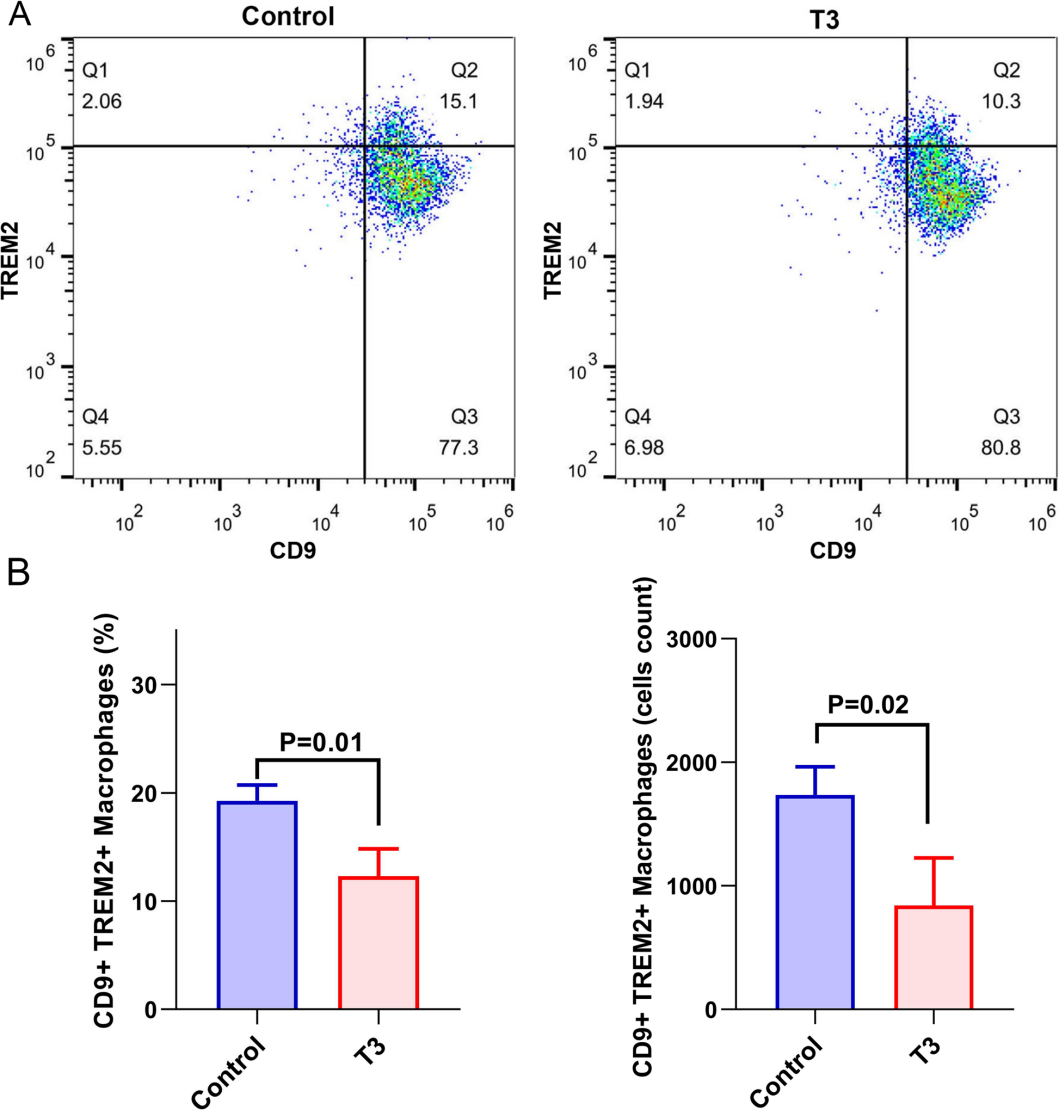
T3 inhibited the profibrotic TREM2 ^+^CD9^+^macrophage(**A**. Flow cytometry showing the proportion of TREM2^+^CD9^+^ macrophages in the T3 intervention group versus the control group; **B**. Difference in the TREM2^+^CD9^+^ macrophages percentage (left) and cell counts (right) between groups.)

A study in 2019 identified a subpopulation of profibrotic TREM2^+^CD9^+^ macrophages, which had a key role in the progression of liver fibrosis [30]. In our in-vitro experiment, CD9^+^TREM2^+^ macrophage subpopulation was found to be significantly decreased in the T3 intervention group by flow cytometry, and the cell count of CD9^+^TREM2^+^ macrophages were also significantly decreased (Fig. 4-A and B).

## Discussion

The relationship between fT3 level and liver fibrosis is still unclear. Therefore, we performed a cross-sectional study to evaluate this relationship. This study included 2072 T2DM patients and found that fT3 levels in T2DM patients were significantly and negatively correlated with the severity of liver fibrosis, and lower fT3 level within the normal range was an independent risk factor of liver fibrosis.

The association between THs and liver fibrosis is unclear and has become a hot topic of research. A recent meta-analysis included 14 cohort studies of 17,301 patients with biopsy-proven NAFLD and showed that the 5- and 10-year all-cause mortality rates for stage 0–2 fibrosis were 3.3% and 7.7%, respectively, while the 5- and 10-year all-cause mortality rates for stage 4 fibrosis were as high as 20.6% and 41.5%. The risk of liver-related mortality was found to increase exponentially with the progression of fibrosis, with a 15.1-fold increased risk of death for stage 4 fibrosis compared with stage 1 fibrosis [31]. Another meta-analysis included 13 studies and a total of 4428 patients with biopsy-proven NAFLD, and found that all-cause mortality and liver-related mortality were 3.42 and 11.13 times higher in patients with stage 4 fibrosis than in patients without fibrosis [32]. All of these data suggest that the risk of all-cause and liver-related mortality in patients with NAFLD increases significantly with the severity of fibrosis. Therefore, fibrosis is a key determinant of clinical prognosis in patients with NAFLD and requires timely screening and treatment. It has also been shown that T2DM patients have a higher prevalence of liver fibrosis compared to non-T2DM patients [33], and T2DM significantly increases the risk of liver fibrosis in overweight or obese individuals, suggesting that screening for liver fibrosis is important in adults with T2DM [17].

Some observational studies explored the influence of THs on liver fibrosis. A study showed that the severity of fibrosis in NAFLD patients was negatively correlated with the levels of fT3, and low fT3 levels were an independent risk factor for severe liver fibrosis [34]. Another study showed that fT3/fT4 ratio was higher in NASH patients with cirrhosis than in healthy controls and could be used as a non-invasive marker of liver fibrosis severity in NASH patients [35]. A cross-sectional study collected liver biopsies from 85 patients with different stages of NASH who underwent bariatric surgery and found that THRB mRNA expression levels were significantly negatively correlated with NAFLD severity, suggesting a lower response in the liver to THs during disease progression [36]. A case-control study included 29 patients with cirrhosis and 50 healthy controls showed that fT4 and fT3 levels were significantly lower in patients with cirrhosis than in healthy controls even though fT4 and fT3 levels were within normal range, and fT3 levels were negatively correlated with the severity of liver dysfunction [23]. Another single-center study found a significant negative correlation between fT3 levels and severity of liver fibrosis [26]. However, a cross-sectional study showed a positive correlation between fT3 levels and the severity of liver fibrosis in patients with NAFLD [34]. Therefore, the relationship between fT3 levels and liver fibrosis is still inconclusive. The findings from our study suggested that lower fT3 level within the normal range was an independent risk factor for the progression of liver fibrosis. However, most studies including our present study used retrospective design and could not provide a precise estimate on the influence of THs on liver fibrosis owing to the impact of confounding factors. Therefore, the causal relationship between THs and liver fibrosis needs to be evaluated in depth with prospective cohort studies in the future.

The relationship between fT3 levels and liver fibrosis in patients without T2DM had been studied in several studies. A recent meta-analysis included 18 case-control studies with a total of 3336 participants and analyzed the correlation between thyroid hormones and the severity of cirrhosis in patients [37]. The results showed that fT3 and fT4 levels decreased and TSH levels increased in patients with cirrhosis and that fT3 levels were negatively correlated with the Child-Pugh score, a measure of the severity of cirrhotic dysfunction [37]. A study of a prognostic prediction model for patients with chronic liver failure showed that fT3 was a protective factor for prognosis in patients with Hepatitis B virus-related acute-on-chronic liver failure (HBV-ACLF) [38]. It was also found that fT3 levels were significantly lower in both cirrhotic patients and HCC patients than in healthy controls [39]. Some studies have shown a decrease in fT3 levels with increasing age. There is a modest decline in the fT3 levels in older persons compared with those non-old individuals, and an increased prevalence of hypothyroidism in the elderly population [40, 41]. The changes of fT3 levels in T2DM patients compared with healthy populations had been explored by some studies and the findings suggested lower fT3 levels in T2DM patients than in non-T2DM controls [42, 43].

Based on the results of the above studies, we should promptly screen T2DM and NAFLD patients for thyroid function especially fT3 levels. If NAFLD patients have hypothyroidism or if patients have normal thyroid function but low fT3 levels, they may be at high risk of liver fibrosis progression. To reduce the risk of liver fibrosis among those patients, THs replacement therapy may be considered. A study found that low-dose T4 was a treatment for hepatic steatosis and early stage of NASH, while low-dose T3 or thyroxine analogs may be more effective in the advanced stages of NASH [44]. Recently, THRβ agonists were found to be effective for the prevention and treatment of hepatic steatosis and NASH [45]. For example, Resmetirom (MGL-3196), a THRβ agonist, significantly improved hepatic steatosis in NASH patients and ameliorated the progression of liver fibrosis in mice with advanced NASH without affecting body weight [46, 47]. In a mouse model of cirrhosis due to severe NASH, administration of T3 reduced hepatic triglycerides, hepatic inflammation and liver fibrosis [48]. In addition, we assessed the impact of hypothyroidism on the progression of liver fibrosis in mice model of NASH. The results proved that hypothyroidism mice had more severe of liver fibrosis than those mice with normal thyroid function.

Liver fibrosis is the result of excessive repair of chronic liver injury, mainly manifested by excessive production and deposition of extracellular matrix (ECM) [49]. Hepatic stellate cells (HSCs) are involved in the formation of fibrosis during the progression of liver disease, and activated HSCs are the main source of ECM in liver fibrosis [50]. Under physiological conditions, HSCs are in a resting state and regulate the homeostasis of ECM; while during the progression of liver fibrosis, factors such as pro-fibrotic factors released from inflammatory cells activate HSCs, which convert them into myofibroblasts with proliferative and contractile functions and synthesize excessive collagen fibers, resulting in massive ECM and fibrous tissue formation [50]. Activation of HSCs involves multiple factors, such as platelet-derived growth factor (PDGF), transforming growth factor β (TGF-β) and TNF-α can induce the activation of HSCs [51–54]. However, the pathogenic mechanisms causing the progression of liver fibrosis are not fully understood and need to be studied in depth.

The mechanism underlying the influence of fT3 on liver fibrosis has not been elucidated. A study found that T3 ameliorated the progression of liver inflammation and fibrosis in NASH mice by restoring autophagy and mitochondrial biosynthesis, thereby increasing fatty acid β-oxidation [48]. FT3 in the liver can bind to thyroid hormone receptors (TRs) α and TRβ, which can further reduce intrahepatic triglyceride and cholesterol levels and restores mitochondrial function in hepatocytes [55]. Alonso-Merino et al. reported that collagen spontaneously accumulated in the liver of TRs knockout mice, and T3 supplementation inhibited CCl4-induced liver fibrosis [55]. They proposed that T3 could antagonize TGF-β-mediated liver fibrosis progression by inhibiting SMAD transcriptional activity [55]. It was also found that T3 may ameliorate the inflammatory response and progression of cirrhosis in mice with alcoholic fatty liver by negatively regulating the NLRP3 signaling pathway [56]. However, the mechanisms underlying the role of FT3 in liver fibrosis are still largely elusive, and need to be studied in future. In our study, we found that T3 could inhibit the profibrotic macrophage TREM2^+^CD9^+^ macrophage, which had been identified an important player in the progression of liver fibrosis.

The present study is a cross-sectional study and is unable to assess the causality relationship between lower fT3 level and progression of liver fibrosis. Cohort studies with the ability of assessing the causality relationship are needed to adequately evaluate lower fT3 level is a risk factor for the progression of liver fibrosis. Besides, the relationship between lower fT3 level and liver fibrosis in healthy individuals or patients with non-T2DM diseases still need to be explored in additional studies.

In summary, the findings from this study proved an inverse correlation between T3 level and the severity of liver fibrosis, and lower fT3 level within the normal range was an independent risk factor for severe liver fibrosis. Routine monitoring of serum fT3 levels in T2DM or NAFLD patients may be important for identifying individuals with advanced liver fibrosis or those at high risk of fibrosis progression.

## Supplementary Information

**Additional file 1:** **Supplementary Table 1.** Methods of assessing the severity of liver fibrosis. **Supplementary Table 2.** Differences in the clinical features among those groups classified by BARD Score. **Supplementary Table 3.** Differences in NFS score, FIB-4 score and BARD score among T2DM patients grouped by fT3 levels.

## Data Availability

The datasets generated or analyzed during the current study are not publicly available due to data sharing policies but are available from the corresponding author on reasonable request.

## Abbreviations

NAFLD: Non-alcoholic fatty liver disease
T2DM: Type 2 diabetes mellitus
MetS: Metabolic syndrome
NAFL: Non-alcoholic fatty liver
NASH: Non-alcoholic steatohepatitis
ADA: American Diabetes Association
THs: Thyroid hormones
T4: Thyroxine
T3: Triiodothyronine
fT3: free triiodothyronine
FPG: Fasting plasma glucose
TG: Triglycerides
TC: Total cholesterol
TG: Triacylglyceride
HDL-C: High density lipoprotein cholesterol
LDL-C: Low density lipoproteincholesterol
ALT: Alanine aminotransferase
AST: Aspartate aminotransferase
BUN: Blood urea nitrogen
sCr: Serum creatinine
SUA: Serum uric acid
TBIL: Total bilirubin
fT4: free thyroxine
TSH: Thyroid-stimulating hormone
BMI: Body mass index
NFS: NAFLD fibrosis score
FIB-4: Fibrosis index based on the 4 factors
BARD: BARD score
SD: Standard deviation

## References

[1] McPherson S, Armstrong MJ, Cobbold JF, Corless L, Anstee QM, Aspinall RJ (2022). Quality standards for the management of non-alcoholic fatty liver disease (NAFLD): consensus recommendations from the British Association for the study of the liver and british Society of Gastroenterology NAFLD Special Interest Group. Lancet Gastroenterol Hepatol..

[2] Santos-Laso A, Gutierrez-Larranaga M, Alonso-Pena M, Medina JM, Iruzubieta P, Arias-Loste MT (2021). Pathophysiological mechanisms in non-alcoholic fatty liver disease: from drivers to targets. Biomedicines..

[3] Devi J, Raees A, Butt AS (2022). Redefining non-alcoholic fatty liver disease to metabolic associated fatty liver disease: is this plausible?. World J Hepatol.

[4] Younossi ZM, Koenig AB, Abdelatif D, Fazel Y, Henry L, Wymer M (2016). Global epidemiology of nonalcoholic fatty liver disease-Meta-analytic assessment of prevalence, incidence, and outcomes. Hepatology.

[5] Chalasani N, Younossi Z, Lavine JE, Charlton M, Cusi K, Rinella M (2018). The diagnosis and management of nonalcoholic fatty liver disease: practice guidance from the American Association for the study of Liver Diseases. Hepatology.

[6] Stefan N, Cusi K (2022). A global view of the interplay between non-alcoholic fatty liver disease and diabetes. Lancet Diabetes Endocrinol.

[7] Lee CH, Lui DT, Lam KS (2022). Non-alcoholic fatty liver disease and type 2 diabetes: an update. J Diabetes Investig.

[8] Portillo-Sanchez P, Bril F, Maximos M, Lomonaco R, Biernacki D, Orsak B (2015). High prevalence of nonalcoholic fatty liver disease in patients with type 2 diabetes Mellitus and normal plasma aminotransferase levels. J Clin Endocrinol Metab.

[9] Targher G, Bertolini L, Padovani R, Rodella S, Tessari R, Zenari L (2007). Prevalence of nonalcoholic fatty liver disease and its association with cardiovascular disease among type 2 diabetic patients. Diabetes Care.

[10] Ortiz-Lopez C, Lomonaco R, Orsak B, Finch J, Chang Z, Kochunov VG (2012). Prevalence of prediabetes and diabetes and metabolic profile of patients with nonalcoholic fatty liver disease (NAFLD). Diabetes Care.

[11] Younossi ZM, Gramlich T, Matteoni CA, Boparai N, McCullough AJ (2004). Nonalcoholic fatty liver disease in patients with type 2 diabetes. Clin Gastroenterol Hepatol.

[12] Wang C, Wang X, Gong G, Ben Q, Qiu W, Chen Y (2012). Increased risk of hepatocellular carcinoma in patients with diabetes mellitus: a systematic review and meta-analysis of cohort studies. Int J Cancer.

[13] American Diabetes Association (2020). Comprehensive Medical Evaluation and Assessment of Comorbidities: Standards of Medical Care in Diabetes-2020. Diabetes Care..

[14] Dulai PS, Singh S, Patel J, Soni M, Prokop LJ, Younossi Z (2017). Increased risk of mortality by fibrosis stage in nonalcoholic fatty liver disease: systematic review and meta-analysis. Hepatology.

[15] Armstrong MJ, Hazlehurst JM, Parker R, Koushiappi E, Mann J, Khan S (2014). Severe asymptomatic non-alcoholic fatty liver disease in routine diabetes care; a multi-disciplinary team approach to diagnosis and management. QJM.

[16] Williamson RM, Price JF, Hayes PC, Glancy S, Frier BM, Johnston GI (2012). Prevalence and markers of advanced liver disease in type 2 diabetes. QJM.

[17] Barb D, Repetto EM, Stokes ME, Shankar SS, Cusi K (2021). Type 2 diabetes mellitus increases the risk of hepatic fibrosis in individuals with obesity and nonalcoholic fatty liver disease. Obes (Silver Spring).

[18] Salvatore D, Porcelli T, Ettleson MD, Bianco AC (2022). The relevance of T3 in the management of hypothyroidism. Lancet Diabetes Endocrinol.

[19] Mullur R, Liu YY, Brent GA (2014). Thyroid hormone regulation of metabolism. Physiol Rev.

[20] Bano A, Chaker L, Plompen EP, Hofman A, Dehghan A, Franco OH (2016). Thyroid function and the risk of nonalcoholic fatty liver disease: the Rotterdam Study. J Clin Endocrinol Metab.

[21] He W, An X, Li L, Shao X, Li Q, Yao Q (2017). Relationship between Hypothyroidism and non-alcoholic fatty liver disease: a systematic review and Meta-analysis. Front Endocrinol (Lausanne).

[22] Kim D, Vazquez-Montesino LM, Escober JA, Fernandes CT, Cholankeril G, Loomba R (2020). Low thyroid function in nonalcoholic fatty liver disease is an independent predictor of all-cause and Cardiovascular Mortality. Am J Gastroenterol.

[23] Vincken S, Reynaert H, Schiettecatte J, Kaufman L, Velkeniers B (2017). Liver cirrhosis and thyroid function: friend or foe?. Acta Clin Belg.

[24] Tas A, Koklu S, Beyazit Y, Kurt M, Sayilir A, Yesil Y (2012). Thyroid hormone levels predict mortality in intensive care patients with cirrhosis. Am J Med Sci.

[25] Huang X, Jiang S, Fan X, Jiang Y, Wu L, Li F (2020). Low-free triiodothyronine is associated with poor prognosis of portal hypertension in cirrhosis. Eur J Gastroenterol Hepatol.

[26] Manka P, Bechmann L, Best J, Sydor S, Claridge LC, Coombes JD (2019). Low free triiodothyronine is Associated with Advanced Fibrosis in patients at high risk for nonalcoholic steatohepatitis. Dig Dis Sci.

[27] Kim D, Kim W, Joo SK, Bae JM, Kim JH, Ahmed A (2018). Subclinical Hypothyroidism and Low-Normal Thyroid Function Are Associated With Nonalcoholic Steatohepatitis and Fibrosis. Clin Gastroenterol Hepatol.

[28] Rigor J, Diegues A, Presa J, Barata P, Martins-Mendes D (2022). Noninvasive fibrosis tools in NAFLD: validation of APRI, BARD, FIB-4, NAFLD fibrosis score, and Hepamet fibrosis score in a portuguese population. Postgrad Med.

[29] Sun W, Cui H, Li N, Wei Y, Lai S, Yang Y (2016). Comparison of FIB-4 index, NAFLD fibrosis score and BARD score for prediction of advanced fibrosis in adult patients with non-alcoholic fatty liver disease: a meta-analysis study. Hepatol Res.

[30] Ramachandran P, Dobie R, Wilson-Kanamori JR, Dora EF, Henderson BEP, Luu NT (2019). Resolving the fibrotic niche of human liver cirrhosis at single-cell level. Nature.

[31] Ng CH, Lim WH, Hui Lim GE, Hao Tan DJ, Syn N, Muthiah MD, et al. Mortality outcomes by Fibrosis Stage in nonalcoholic fatty liver disease: a systematic review and Meta-analysis. Clin Gastroenterol Hepatol 2022. [Online ahead of print] 10.1016/j.cgh.2022.04.014 PMC10792524 35513235

[32] Taylor RS, Taylor RJ, Bayliss S, Hagstrom H, Nasr P, Schattenberg JM (2020). Association between Fibrosis Stage and Outcomes of patients with nonalcoholic fatty liver disease: a systematic review and Meta-analysis. Gastroenterology.

[33] Chen J, Hu P, Wang Y, Zhu Z (2022). Association between type 2 diabetes status and prevalence of liver steatosis and fibrosis among adults aged >/= 40 years. BMC Endocr Disord.

[34] Guo W, Qin P, Li XN, Wu J, Lu J, Zhu WF (2021). Free triiodothyronine is Associated with hepatic steatosis and liver stiffness in euthyroid chinese adults with non-alcoholic fatty liver disease. Front Endocrinol (Lausanne).

[35] Turker F, Oral A, Sahin T, Turker BC, Kocak E, Ataoglu HE (2021). Does the FT3-to-FT4 ratio easily predict the progression of NAFLD and NASH cirrhosis?. J Int Med Res.

[36] Krause C, Grohs M, El Gammal AT, Wolter S, Lehnert H, Mann O (2018). Reduced expression of thyroid hormone receptor beta in human nonalcoholic steatohepatitis. Endocr Connect.

[37] Liu C, Li L, Zeng L (2022). The Clinical Value of Thyroid Hormone Levels and Correlation with Severity of Liver Cirrhosis. Comput Intell Neurosci..

[38] Zhang J, Chen Y, Duan Z (2022). Development of a FT3-related prognostic model for patients with hepatitis B virus-related acute-on-chronic liver failure. Bioengineered.

[39] Sahin T, Oral A, Turker F, Kocak E (2020). Can hypothyroidism be a protective factor for hepatocellular carcinoma in cirrhosis?. Med (Baltim).

[40] Santos Palacios S, Llavero Valero M, Brugos-Larumbe A, Diez JJ, Guillen-Grima F, Galofre JC (2018). Prevalence of thyroid dysfunction in a large Southern European Population. Analysis of modulatory factors. The APNA study. Clin Endocrinol (Oxf).

[41] Kussmaul T, Greiser KH, Haerting J, Werdan K, Thiery J, Kratzsch J (2014). Thyroid analytes TSH, FT3 and FT4 in serum of healthy elderly subjects as measured by the Roche modular system: do we need age and gender dependent reference levels?. Clin Lab.

[42] Li Y, Yi M, Deng X, Li W, Chen Y, Zhang X (2022). Evaluation of the thyroid characteristics and correlated factors in hospitalized patients with newly diagnosed type 2 diabetes. Diabetes Metab Syndr Obes.

[43] Gu L, Yang J, Gong Y, Ma Y, Yan S, Huang Y (2021). Lower free thyroid hormone levels are associated with high blood glucose and insulin resistance; these normalize with metabolic improvement of type 2 diabetes. J Diabetes.

[44] Bruinstroop E, Dalan R, Cao Y, Bee YM, Chandran K, Cho LW (2018). Low-dose levothyroxine reduces intrahepatic lipid content in patients with type 2 diabetes Mellitus and NAFLD. J Clin Endocrinol Metab.

[45] Sinha RA, Bruinstroop E, Singh BK, Yen PM (2019). Nonalcoholic fatty liver Disease and Hypercholesterolemia: roles of thyroid hormones, metabolites, and agonists. Thyroid.

[46] Harrison SA, Bashir MR, Guy CD, Zhou R, Moylan CA, Frias JP (2019). Resmetirom (MGL-3196) for the treatment of non-alcoholic steatohepatitis: a multicentre, randomised, double-blind, placebo-controlled, phase 2 trial. Lancet.

[47] Kannt A, Wohlfart P, Madsen AN, Veidal SS, Feigh M, Schmoll D (2021). Activation of thyroid hormone receptor-beta improved disease activity and metabolism independent of body weight in a mouse model of non-alcoholic steatohepatitis and fibrosis. Br J Pharmacol.

[48] Zhou J, Tripathi M, Ho JP, Widjaja AA, Shekeran SG, Camat MD (2022). Thyroid hormone decreases hepatic steatosis, inflammation, and fibrosis in a Dietary Mouse Model of Nonalcoholic Steatohepatitis. Thyroid.

[49] Wells RG, Schwabe RF (2015). Origin and function of myofibroblasts in the liver. Semin Liver Dis.

[50] Trautwein C, Friedman SL, Schuppan D, Pinzani M (2015). Hepatic fibrosis: Concept to treatment. J Hepatol.

[51] Friedman SL (2000). Molecular regulation of hepatic fibrosis, an integrated cellular response to tissue injury. J Biol Chem.

[52] Hellerbrand C, Stefanovic B, Giordano F, Burchardt ER, Brenner DA (1999). The role of TGFbeta1 in initiating hepatic stellate cell activation in vivo. J Hepatol.

[53] Tsuchida T, Friedman SL (2017). Mechanisms of hepatic stellate cell activation. Nat Rev Gastroenterol Hepatol.

[54] Kim BM, Abdelfattah AM, Vasan R, Fuchs BC, Choi MY (2018). Hepatic stellate cells secrete Ccl5 to induce hepatocyte steatosis. Sci Rep.

[55] Alonso-Merino E, Martin Orozco R, Ruiz-Llorente L, Martinez-Iglesias OA, Velasco-Martin JP, Montero-Pedrazuela A (2016). Thyroid hormones inhibit TGF-beta signaling and attenuate fibrotic responses. Proc Natl Acad Sci U S A.

[56] Dong X, Yang H, Li C, Liu Q, Bai Q, Zhang Z (2018). Triiodothyronine alleviates alcoholic liver disease injury through the negative regulation of the NLRP3 signaling pathway. Exp Ther Med.

