# Supplementary material for: The correlation between triiodothyronine and the severity of liver fibrosis
Source: BMC Endocr Disord. 2022 Dec 12;22:313. doi: 10.1186/s12902-022-01228-8 (PMC9743744; doi:10.1186/s12902-022-01228-8)
Supplement: Supplementary file 1 — Additional file 1: Supplementary Table 1. Methods of assessing the severity of liver fibrosis. Supplementary Table 2. Differences in the clinical features among those groups classified by BARD Score. Supplementary Table 3. Differences in NFS score, FIB-4 score and BARD score among T2DM patients grouped by fT3 levels. [file 12902_2022_1228_MOESM1_ESM.docx]

**Supplementary table 1 Methods of assessing the severity of liver fibrosis**

| Items | Calculation formula |
| --- | --- |
| NFS | −1.675 + (0.037 × Age[years]) + (0.094 × BMI) + (1.13 × IFG/diabetes [yes=1, no=0]) + (0.99 × AST/ALT) - (0.013 × platelet[10^9^/l]) - (0.66 × albumin[g/dl]) |
| FIB-4 | Age[years] × AST[U/L]) / (platelet [10^9^] ×√ALT[U/L] |
| BARD | Total score scale: 0-4  BMI ≥ 28 kg/m^2^ = 1 point  AST to ALT Ratio (AAR) ≥ 0.8 = 2 points  Diabetes mellitus = 1 point |

(NFS, NAFLD fibrosis score; FIB-4, fibrosis index based on the 4 factors; BARD, BARD score; BMI, body mass index; IFG, impaired fasting glucose; AST, aspartate aminotransferase; ALT, alanine aminotransferase)

**Supplementary table 2 Differences in the clinical features among those groups classified by BARD Score**

| Parameters | 1/2 | 3/4 | P values |
| --- | --- | --- | --- |
| Number | 864 | 1208 |  |
| Gender (Male, %) | 588(68.1%) | 681(56.4%) | <0.001 |
| Age (Year, Mean ± SD) | 51.45(13.79) | 58.77(13.01) | <0.001 |
| Hypertension (%) | 326(37.7%) | 572(47.4%) | <0.001 |
| BMI (kg/M2, Mean ± SD) | 25.53(4.19) | 24.10(3.79) | 0.010 |
| Obesity (%) | 189(21.9%) | 154(12.7%) | <0.001 |
| Duration (Year, Mean ± SD) | 7.84(2.34) | 8.06(3.56) | 0.210 |
| TSH (mIU/L, Mean ± SD) | 1.67(0.87) | 1.76(0.89) | <0.001 |
| FT4 (pmol/L, Mean ± SD) | 16.92(2.25) | 16.59(2.17) | 0.002 |
| FT3 (pmol/L, Mean ± SD) | 4.70(0.53) | 4.49(0.50) | <0.001 |

**(**BMI, body mass index; TSH, thyroid stimulating hormone; fT3, free triiodothyronine; fT4, free thyroxine)

**Supplementary table 3 Differences in NFS score, FIB-4 score and BARD score among T2DM patients grouped by fT3 levels**

| Parameters | Q1 | Q2 | Q3 | P values |
| --- | --- | --- | --- | --- |
| Number | 696 | 689 | 687 |  |
| Gender (Male, %) | 323(46.4%) | 414(60.1%) | 532(77.4%) | <0.001 |
| Age (Year, Mean ± SD) | 59.94(12.97) | 56.54(13.06) | 50.62(13.79) | <0.001 |
| Hypertension (%) | 340(48.9%) | 301(43.7%) | 257(37.4%) | <0.001 |
| BMI (kg/M2, Mean ± SD) | 23.97(3.73) | 24.77(4.22) | 25.36(3.99) | <0.001 |
| Obesity (%) | 90(12.9%) | 114(16.5%) | 139(20.2%) | 0.001 |
| Duration (Year, Mean ± SD) | 8.40(3.99) | 7.91(2.80) | 7.59(2.22) | <0.001 |
| TSH (mIU/L, Mean ± SD) | 1.74(0.87) | 1.76(0.94) | 1.68(0.83) | 0.254 |
| fT4 (pmol/L, Mean ± SD) | 16.21(2.24) | 16.62(2.04) | 17.36(2.19) | <0.001 |
| NAFLD fibrosis score | -1.00(1.28) | -1.13(1.25) | -1.49(1.15) | <0.001 |
| FIB-4 score | 1.21(0.99) | 1.12(0.75) | 0.96(0.58) | <0.001 |
| BARD score | 2.47(0.98) | 2.39(1.01) | 2.14(1.01) | <0.001 |

**(**BMI, body mass index; TSH, thyroid stimulating hormone; fT4, free thyroxine)
